# *Ochyronemus*, a New Genus of the Tarsonemid Tribe Pseudotarsonemoidini (Acari: Heterostigmatina) from Mexico

**DOI:** 10.3390/insects16010046

**Published:** 2025-01-06

**Authors:** Wojciech Ł. Magowski, Jose M. Rezende, Ronald Ochoa

**Affiliations:** 1 Department of Animal Taxonomy and Ecology, Adam Mickiewicz University, Uniwersytetu Poznańskiego 6, 61-614 Poznań, Poland; 2 Department of Zoology and Botany, Institute of Biosciences, Humanities and Exact Sciences (IBILCE), São Paulo State University (UNESP), Campus São José do Rio Preto, São Paulo 15054-000, Brazil; jmrezende@live.com; 3 Systematic Entomology Laboratory, Agricultural Research Service, United States Department of Agriculture, Beltsville, MD 20705, USA; ron.ochoa@ars.usda.gov

**Keywords:** tarsonemid mites, bark beetles, galleries, wood boring insects

## Abstract

A new genus for the tribe Pseudotarsonemoidini of the family Tarsonemidae is established in order to accommodate undescribed mites associated with bark beetles in Mexico. The new, monotypic genus *Ochyronemus*, with the type species *O. jaliscoe* being diagnostically and phylogenetically closest to the derived *Pseudotarsonemoides*-*Tarsobisulcus* cluster of the Pseudotarsonemoidini, possesses an intermediate position between these two genera. Details of the morphology related to its genus-level affiliation, primarily tibiotarsal I claw and leg setation, are compared in the context of the other genera of the tribe.

## Simple Summary

*Ochyronemus jaliscoe* gen. n., sp. n. is described and illustrated. Its morphology rendering a genus-level affiliation is discussed. An updated key to genera of the tribe *Pseudotarsonemoidini* is supplied.

## 1. Introduction

Tarsonemidae (Acari: Prostigmata) is a large mite family composed of more than 40 genera with a worldwide distribution (see [1,2,3,4,5,6,7]). It is a highly diverse taxonomic assemblage with a wide range of habitats and feeding strategies, e.g., algivory, mycophagy, parasitism, parasitoidism, phytophagy, and egg predation [8]. Even fungivorous soil tarsonemids can reach high levels of diversity in a single locality [9]. In addition, the family has a considerable number of complex associations with plants and various animal groups, especially insects [10]. Among those associations, phoresy is a relationship extensively documented with tarsonemid mites [6,8]. Several species- and genus- level taxa (e.g., *Pseudotarsonemus* Lindquist, 1986 and *Chaetotarsonemus* Beer & Nucifora, 1965) have been discovered only after collecting their specific insect carriers and have not been found elsewhere.

The tribe *Pseudotarsonemidini* Lindquist [8] was established to group five early derivative genera. Four of them are permanent insect associates (*Pseudotarsonemoides* Vitzthum, 1921; *Ununguitarsonemus* Beer & Nucifora, 1965; *Nasutitarsonemus* Beer & Nucifora, 1965; and *Tarsanonychus* Lindquist, 1986) [8] and one (*Polyphagotarsonemus* Beer & Nucifora, 1965) is phytophagous while still using insects (whiteflies, thrips, and aphids) [11] for dispersal. According to the phylogenetic hypotheses of Lindquist [8], the most derivative genus of the tribe is *Pseudotarsonemoides*, a relatively small tarsonemid genus with nine species described ([12,13,14,15,16,17,18]). They have only been recorded in association with Curculionidae (Scolytinae) and Cerambycidae beetles, frequently found to be phoretic on these insects or inhabiting their subcortical galleries. The recent discovery of another genus, *Tarsobisulcus* Khaustov, Fjellberg & Lindquist, 2022 [19], displays an interesting alternate evolutionary path. Unlike *Pseudotarsonemoides* and other kin-genera, *Tarsobisulcus angulomarginis* is uniquely associated with gall midges (Cecidomyidae) on oaks in Norway. The new acari taxon presented here was discovered and announced by one of us (R. Ochoa) during the Annual Meeting of Entomological Society of America (in Louisville, Kentucky, USA) in the year 1996 [20]. However, this did not reach the stage of a formal description. The re-discovery of the specimens in the Canadian National Collections (courtesy of Dr. E. E. Lindquist) led us to conduct further morphological examination, the early results of which were presented during XXXIII Brazilian Congress of Zoology [21].

The new species seems to fit between the pre-existing concept of the *Pseudotarsonemoides* on the one hand and the *Tarsobisulcus* on the other, and hence deserves the establishment of a new taxon named *Ochyronemus* n. gen., which is described below.

## 2. Material and Methods

The material used for the present study is a series of microscopic slide preparations (type and non-type specimens) lent courtesy of the Canadian National Collection of Insects, Arachnids and Nematodes, Ottawa, Ontario, Canada (for further details see section Type material). Examination, measurements, drawings, and microphotographs were mostly executed with an Olympus BX50 phase-contrast microscope supplied with a drawing tube attachment and a digital camera, an Olympus E-5. An Olympus BX51 with differential interference contrast (DIC) and digital camera, Canon EOS 5D Mk. II, was also used for some of the microphotographs. Line drawings were processed with an Inkscape vector graphic software [22]. Micrographs were stacked with PICOLAY software version 2024-04-13 [23] and processed (along with line graphics) with Corel PHOTO-PAINT 2021 version 23.5.0.506.

The following abbreviations are used for institutions where the types mentioned are deposited: CNC (Canadian National Collection of Insects, Arachnids and Nematodes, Ottawa, Ontario, Canada); AMUNC (Natural Collections, Adam Mickiewicz University, Poznań, Poland).

The terminology used herein follows mainly Lindquist [8], except for the gnathosomal setae (*ch* and *su* [24]), and propodosomal and metapodosomal ventral plates (MtP and PrP, respectively [25]). For all structures, measurements are in micrometers (µm). Holotype measurements are given first followed by the spread among five paratypes in parentheses. Tegula length is measured from the level of its base (at trochanters IV) to a posterior margin. Leg setation expresses number of non-solenidial phaneres; numbers of solenidia are separated in parentheses and the sign “+” marks fusion of segments. Indistinct setae *u″* flanking pretarsi II and III are excluded from the count; however, setiform seta Tbt I *s*, small but thick Tbt I *u′*-*u″*, and spine-like Ta II–III *u′* are all included as being well pronounced.

## 3. Results

Family *Tarsonemidae* Canestrini & Fanzago, 1877 [26].

Subfamily Pseudotarsonemoidinae Lindquist, 1986 [8].

Tribe *Pseudotarsonemoidini* Lindquist, 1986 [8].

***Ochyronemus* gen. n.**

Type species: *Ochyronemus jaliscoe* sp. n.

Description: Adult female. Gnathosoma: capsule ca. as long as wide, rounded short ovoid. Palpi short, stout; cheliceral stylets thin but well pronounced, occupying almost half length of gnathosomal capsule. Pharynx fusiform in shape with glandular bodies small, posteriorly appressed.

Idiosoma: obovate in shape. Dorsum: anterior projection of prodorsal shield covering ca. two-thirds of gnathosoma; lateral margins indistinctly emarginated near bothridia level; stigmata openings located closely behind setae *v*_1_; trichobothria *sc*_1_ capitate, obovate. Posterior margin of tergite C concave medially; that of tergite D weakly convex medially. Setae *v*_1_ ca. three-quarters of *sc*_2_ length; dorsal setae *c*_1_, *d* and *f* stiff, indistinctly pointed but remaining ones slender, sharp; *h* ca. four times longer than ventral setae *ps*.

Venter: anteromedial projection of propodosomal plate absent; anterior margin of metapodosomal plate entire, concave but not divided medially. Apodemes 4 weakly developed, extending from apex of trochanters IV to a point mediad setae *3b*; posteromedial apodeme retained only in posterior part; weakly defined. Tegula enlarged, tonguelike, wider at base and overlapping ca. half length of agenital plate; trochanters of legs IV separated by an interval of about twice their widths; setae *1a–4b* stouter from bases to about third of length, more attenuate towards tips.

Leg setation of leg I: 4-4-6(1*φ*)+12(1*ω*); leg II: 3-3-4-6(1*ω*); leg III: 2+3-4-5. Tibiotarsus I almost precisely as long as wide at base; femorogenu IV nearly three times longer than tibiotarsus IV; famulus *k* on tibiotarsus I tapering, bluntly pointed, solenidion *ϕ*_1_ absent; femoral seta *l″* on leg I present; femoral seta *v″* on leg II present; tarsal seta *pl″* on leg II recurved distally.

Differential diagnosis: adult females of *Ochyronemus* are similar to those of both *Pseudotarsonemoides* and *Tarsobisulcus* by large, lobate tegula reaching about half length of aggenital plate, aggenital setae absent, sessile and robust tarsal I claw strongly bent; thin, setiform tarsal I subunguinal seta *s* and tiny tarsal I *ft′* both present. It also shares with *Pseudotarsonemoides* setae *f* smallest on dorsum (vs. *e* smallest in *Tarsobisulcus angulomarginis*), and with *Tarsobisulcus* moderately long and broad pharynx with muscular, thinly sclerotized walls, stigmata located immediately behind the bases of setae *v*_1_ and number of setae on femur I-4, femur II-3, and femorogenu III-5 (vs. well-sclerotized horseshoe-shaped pharynx, stigmata in a distance posteriad of *v*_1_ and number of setae on femur I-3 and II-2 and femorogenu III-4 in *Pseudotarsonemoides*).

*Ochyronemus* n. gen. can be distinguished from both abovementioned genera by the cheliceral stylets occupying ca. half length of gnathosoma (vs. quarter length or less in two others), ventral metapodosomal plate with concave anterior edge medially (vs. bilobed divided medially in *Pseudotarsonemoides* and with triangular lobe projecting forward in *Tarsobisulcus*), by short, stubby tibiotarsus I about as long as wide at base (vs. ca. 1.5× as long as wide at the base in other two genera) and by tibial solenidion *ϕ*_2_ lacking from tibiotarsus I (vs. most commonly present in both other genera).

Males and larvae: unknown.

Etymology: The name of the new genus is based on the Ancient Greek word οχυρό (ochyro = eng. stronghold) because of the robust appearance of the mite’s body.

***Ochyronemus jaliscoe* sp. n.**

Diagnosis: as for the genus.

Description: Female (Figure 1, Figure 2, Figure 3, Figure 4, Figure 5, Figure 6, Figure 7 and Figure 8).

**Figure 1:**
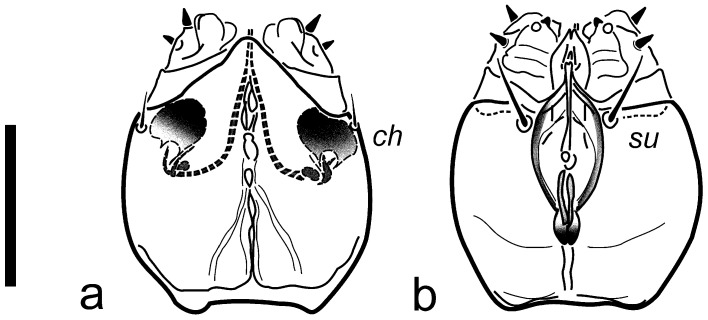
*Ochyronemus jaliscoe* sp. n. female gnathosoma; (**a**) dorsum, (**b**) venter. Scalebar is 20 µm.

Gnathosoma (Figure 1 and Figure 5): capsule ca. as long as wide, rounded but slightly elongate, truncated apically. Pharynx fusiform in shape (Figure 5) somewhat sclerotized in lateral parts (with fine transverse striation visible in few individuals), ca. 0.3× of max. width of gnathosoma and 0.5× of ventral length of the capsule. Paired glandular bodies elongate and relatively thin, not projecting posteriorly outside pharynx outline, though the latter somewhat swollen at the joint with esophageal duct. Setae *ch* short, thin, often indiscernible, ca. 0.4× as long as *su*; both pairs slender, pointed. Postpalpal setae indiscernible. Long and well-defined cheliceral stylets occupying ca. half length of the capsule, cheliceral levers more or less oval in shape (depending on the position), relatively large. Palpi short, cylindrical, convergent, with apparent palptarsal process, small palptibial claw, and two short spinelike setae each.

**Figure 2:**
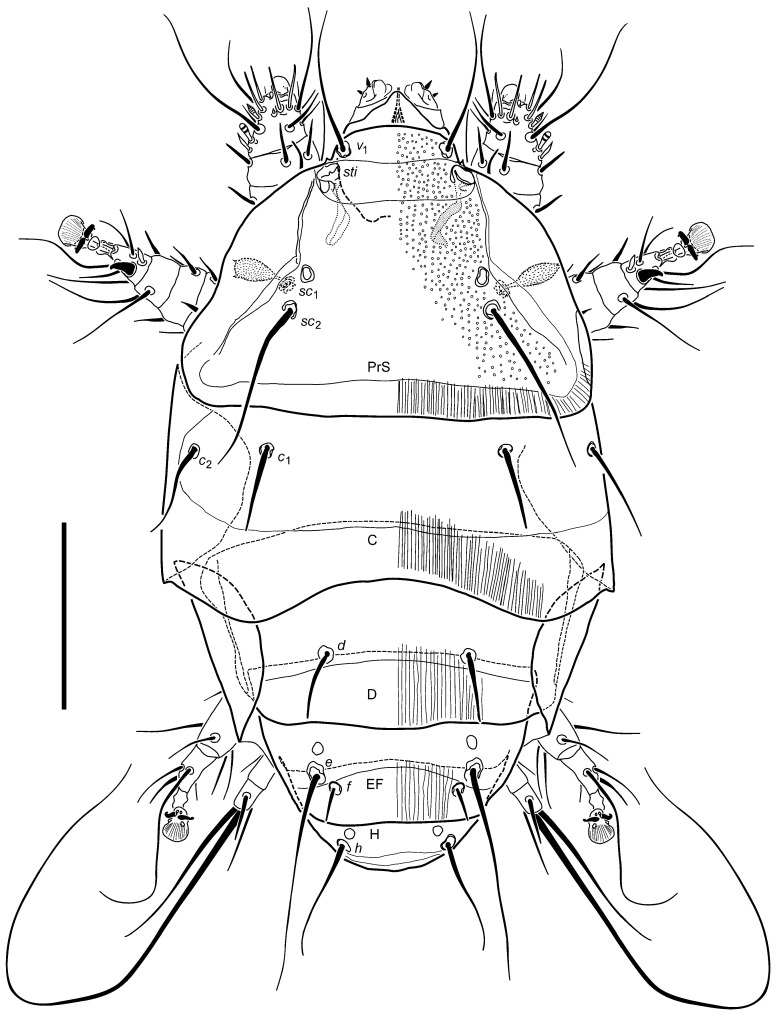
*Ochyronemus jaliscoe* sp. n. female dorsum. Scalebar is 50 µm.

Idiosomal dorsum (Figure 2): average length: 196.8 (SD ± 15.1), width: 105.5 (SD ± 10.2); length/width ratio 1.9×; relative lengths of setae (*v*_1_: *sc*_2_: *c*_2_: *c*_1_: *d*: *e*: *f*: *h*): 1: 1.3: 0.8: 0.7: 0.5: 1.7: 0.3: 0.9.

The shape of prodorsal shield (PrS) near semicircular, on average ca. 1.4× wider than long with posterior edge almost straight. Anterior projection broadly arched (nearly subrectangular), ca. 3× as wide as long (measured as a ratio of distance between stigmae vs. segment from anterial tip to midwith between stigmae). The distance between bases of setae *v*_1_ 0.8× their length. Stigmata located closely posterolaterad *v*_1_, tracheal trunks without apparent atria. Sensilli *sc*_1_ clavate, elongate pilose, with two larger spines apically. Pits *v*_2_ aligned far posteriorly to stigmata, slightly anteromediad sensilli *sc*_1_. Setae *sc*_2_ inserted on posterior half of PrS, protruding beyond posterior edge by 1/4 of their length, located by distance of 1.2× their lengths one to another. Setae *c*_2_ ca. 1.2× as long as *c*_1_, and longer than distance *c*_1_-*c*_2_. Setae *c*_1_ not reaching the posterior edge of C, separated by a distance 2.6× their length each to another. Tergite C with posterior edge distinctly concave. The transverse distance between setae *d* nearly 2.2× their length; *d* with tips reaching posterior edge of D, but not beyond. Posterior margin of D weakly convex in its midsegment. Setae *f* over 6× shorter than *e* and over 3× than *h*, set 3.6× their length apart. Their length subequal to the distance from their base to the edge of tergite EF. Distance between *e* and *f* on each side ca. one-fifth of *f*-*f*. Setae *h* intermediate in length between *d* and *e*; set apart by 0.9× their length. Setae *v*_1_, *sc*_2_, *c*_2_, *e* and *h* slender, attenuate, long, smooth. Setae *c*_1_, *d* and *f* stiffer, shorter than others and bluntly pointed. Dorsal shielding smooth or sculptured with small, uniform, dimples more apparent in anterior and lateral areas of PrS.

**Figure 3:**
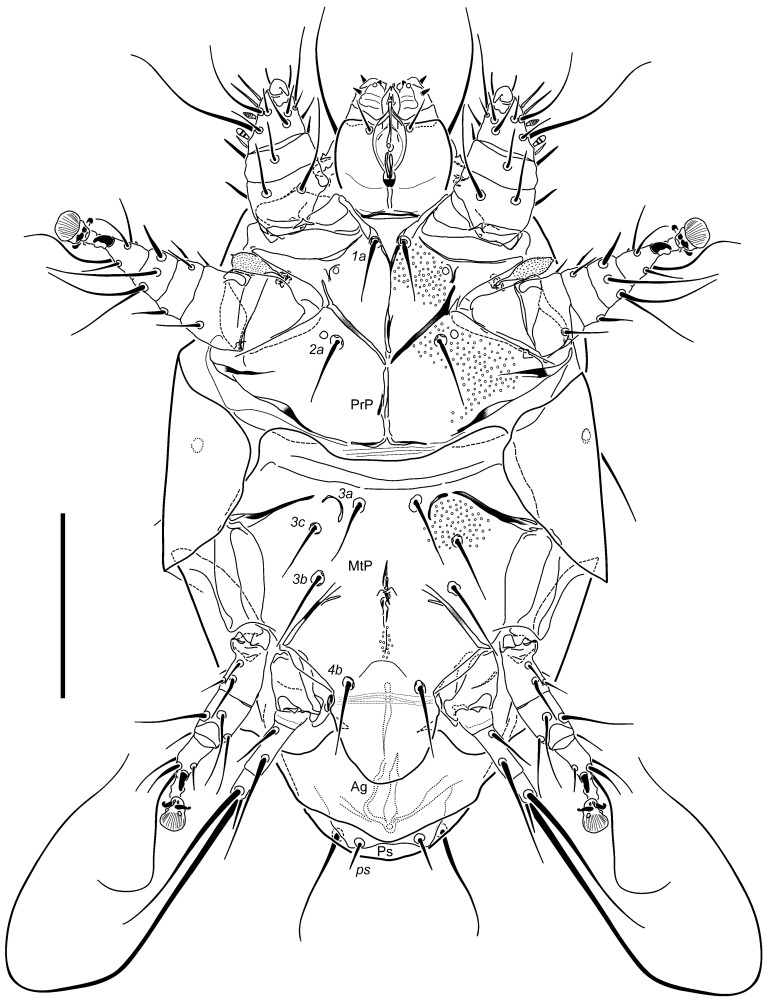
*Ochyronemus jaliscoe* sp. n. female venter. Scalebar is 50 µm.

Idiosomal venter (Figure 3): Apodemes 1 fused to anterior end of anteromedial apodeme. Apodemes 2 reaching anteromedial apodeme posteromedially. The latter extending from junction with apodeme 1 to sejugal apodeme level. Sejugal apodeme more sclerotized in central and lateral segments, each with weaker part along its length. Ridge of ventral propodosomal plate between trochanters I and II indistinctly angular on each side. Setae *1a* separated by distance of 0.4× their lengths. Setae *2a* located posteriad midlengths of apodemes 2 on each side, separated by distance of ca. 1.7× their lengths. Apodemes 3 curved at their anteromedial extremities with terminal segments being separated, extending diagonally from proximity of *3a* bases to anterior tips of trochanters III (and no further); apodemes 4 extending mediad bases of setae *3b* to anterior margins of trochanters IV. Posteromedial apodeme rudimentary, barely discernible beyond level of setae *3b*. Setae *3a* separated by distance of ca. 0.8× their length. Setae *3b* ca. 1.1× longer than *3a*, separated by distance of 1.7× their lengths. Setae *3c* slightly shorter than *3b*, separated by distance of 2.4× their lengths. Setae *4b* separated by a distance about equal to their length. Anterior edge of ventral metapodosomal plate distinctly concave medially. Tegula (Figure 6) lobate rounded at posterior edge, more or less U-shaped in outline, only a little wider than long. Posterior margin of Ag plate broadly triangular, with slightly round apex. Setae *ps* separated by distance of 2× their lengths. All ventral setae slender, attenuate, smooth and pointed, except blunt *ps*. Distal areas of coxosternal fields I and II and proximal of III finely dimpled, besides ventral plates with few or no apparent sculpture.

Soft pleurae connecting tergites C, D, EF, and H of dorsum, and those between pro- and metapodosomal plates and underlying the base of tegula of venter often with dense plication indicating potential for distension.

**Figure 4:**
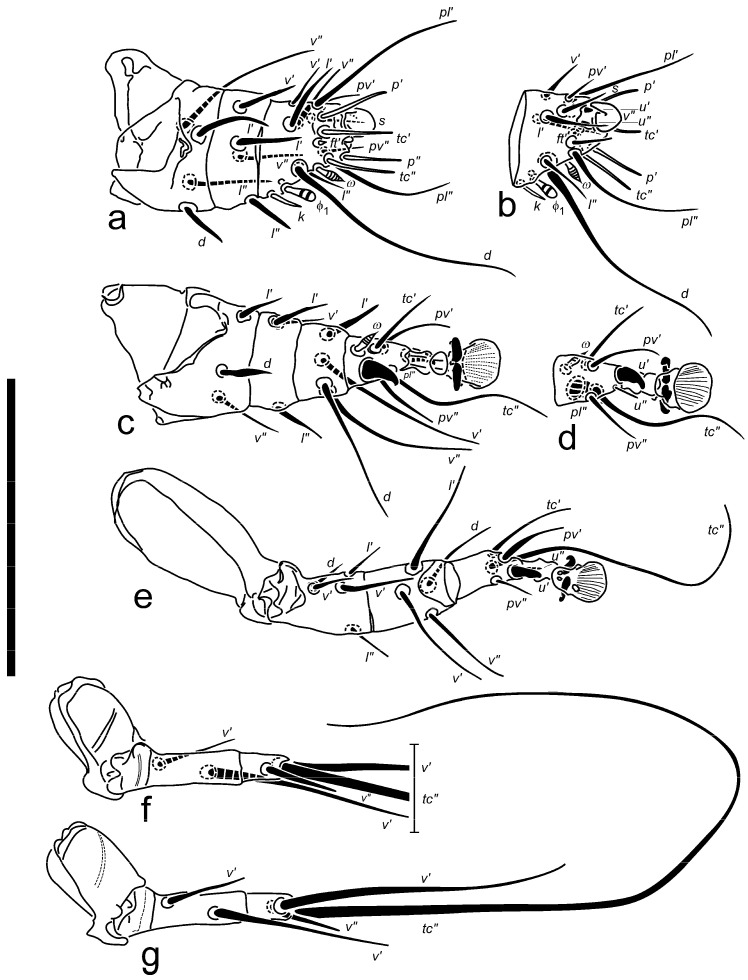
*Ochyronemus jaliscoe* sp. n. female legs (right); (**a**) leg I dorsal aspect, (**b**) tibiotarsus I ventral aspect (paratype), (**c**) leg II dorsal aspect, (**d**) tarsus II ventral aspect, (**e**) leg III ventral aspect, (**f**) leg IV dorsal aspect, (**g**) leg IV ventral aspect. Scalebar is 50 µm.

**Figure 5:**
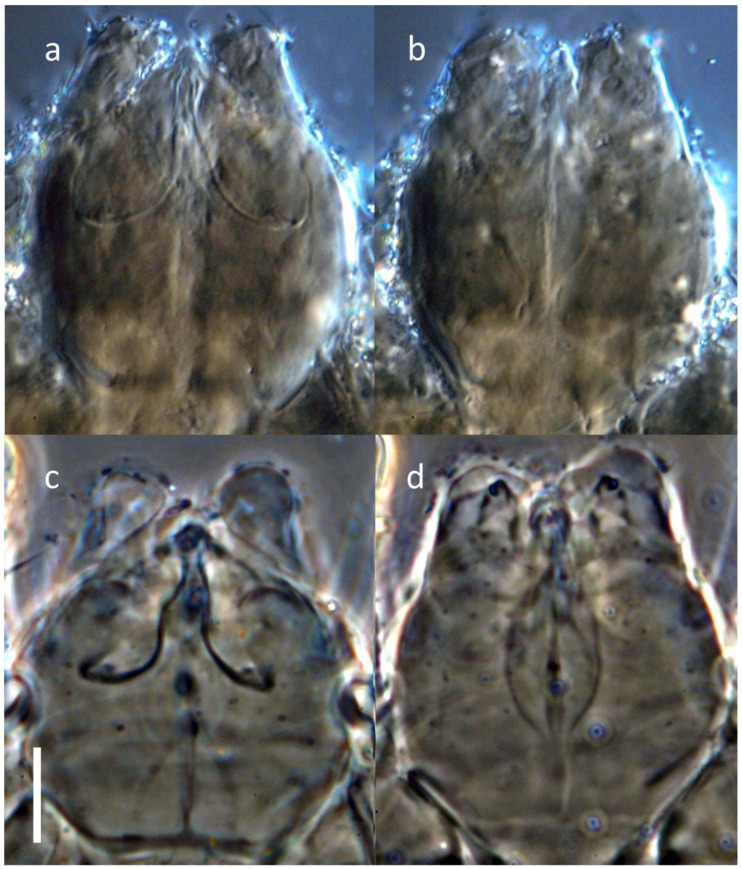
*Ochyronemus jaliscoe* sp. n. female details of gnathosomal morphology; (**a**) dorsal aspect DIC, (**b**) ventral aspect DIC, (**c**) dorsal aspect PhC, (**d**) ventral aspect PhC. Scalebar is 10 µm.

**Figure 6:**
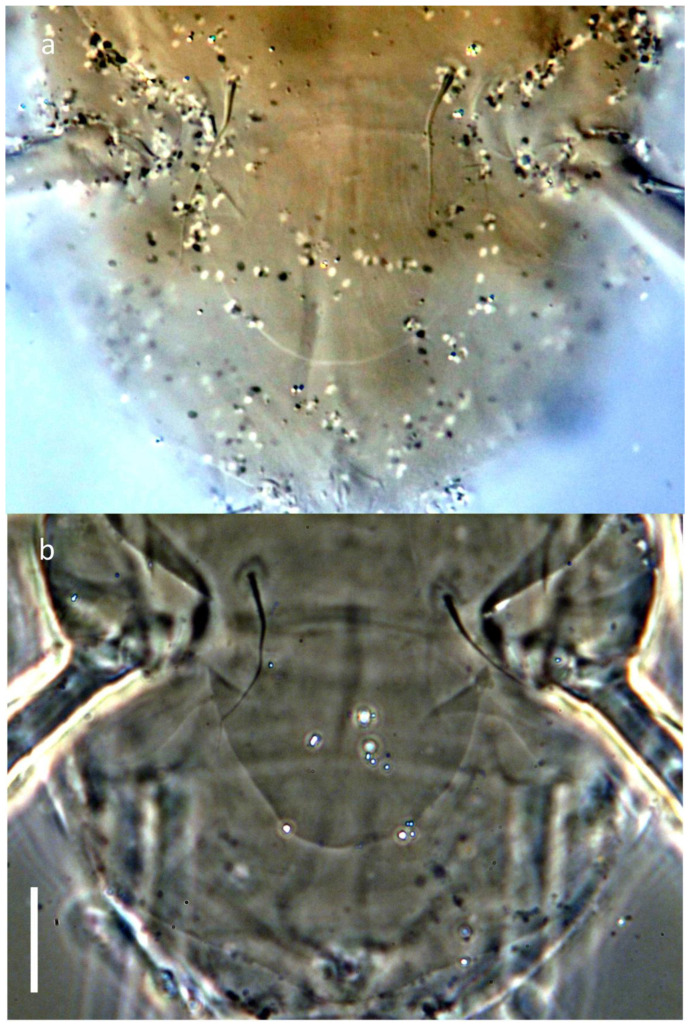
*Ochyronemus jaliscoe* sp. n. female details of posterior metapodosoma and opisthosoma morphology—ventral aspect; (**a**) DIC, (**b**) PhC. Scalebar is 10 µm.

Legs (Figure 4): Proportions of free segments of legs (I: II: III: IV): 1.0: 1.2: 1.1: 0.8. Leg I: tarsal claw large, strongly hooked, inserted almost at apex of tarsus; pretarsal stalk inconspicuous, very short, terminal pad absent. Subunguinal seta *s* setiform, slender, sharply pointed. Unguinal setae *u′* and *u″* on tibiotarsus I very short, spiniform but separate, opposing the extremity of claw. Tibiotarsus short, about as long as wide at the base (without terminal structures included). All four tarsal eupathidia nearly equal in length. Eupathidia *p”* and *p′* located apically, *tc′* and *tc″* subapically (*tc″* may sometimes be displaced in distal 0.3× length of segment). All tarsal setae (*pv′*, *pv″*, *pl′*, and *pl″*) simple, slender, pointed; though both *pl* (esp. *pl″*) well longer than other two. Solenidion *ω* with head rather narrow, striated, with pronounced pointed tip, slightly longer than Ta II *ω*. Solenidion *φ*_2_ absent; *φ*_1_ untypically prominent, bigger than *ω*, but with more swollen and round-ended head. Pointed famulus *k* as long as *φ*_2_. Genual setae *l′*, *l″*, and *v′* stiff, pointed; Ge *l″* more slender. Femoral seta *d* stout pointed; seta *l′* slender; seta *v″* attenuate. Leg II: claws on pretarsus hook-like, thinner than claw I. Seta *u′* spine-like, bent and bluntly tipped; *u″* thin, hardly discernible. Beside solenidion Ta II *ω* spine-like seta *pl″* robust with curved tip, located slightly distally to *ω*, and somewhat bigger than *u′*. Tarsal *tc″* less than 2× longer than *tc′* (shortest on segment), reaching beyond tip of empodium. Tibial *v‘* attenuate, pointed, slightly longer than *d* and *v″*; *l′* shorter, stiff, blunt. All genual setae shorter than tarsal and tibial setae. Femur without lobe; seta *d* stiff, pointed, lanceolate; setae Fe *l′* slender; *v″* simple, attenuate. Leg III: Seta *u′* similar to Ta II *u′*. Tarsal seta *tc″* 3× or more longer than *tc′*, *pv′* and *pv″* (the latter being the shortest on segment). All femorogenual setae slender, Ge *v″* the longest and Fe *d* the shortest. Leg IV: free segments of leg IV slightly shorter than femorogenu and tibia III. Femorogenu almost 3× longer than tibiotarsus. Genual *v′* stouter and ca. 2× longer than femoral *v′*. Tibial *v′* ca. 1.5× longer than free segments of leg IV; tapering, attenuate. Tibial *v″* shortest of all setae on leg IV. Seta Ta *tc″* exceeding over 5× the length of whole leg IV.

Measurements—body and tagmata: length of body: 214 (185–224); length of idiosoma: 204 (180–218); width of idiosoma: 117 (90–115); length of gnathosoma: 35 (33–37); width of gnathosoma: 32 (28–33); length of pharynx: 17 (15–18); width of pharynx: 9 (9–10); *ch*: 4 (3–4); *su*: 8 (7–9). Dorsum—length of PrS: 80 (73–84); width of PrS: 112 (99–116); distance between stigmata proximal: 30 (27–32); distal: 44 (40–46). Lengths of setae: *v*_1_: 36 (30–37); *sc*_1_: 17 (15–17); *sc*_2_: 45 (41–50); *c*_2_: 26 (26–30); *c*_1_: 24 (21–25); *d*: 19 (15–18); *e*: 60 (51–61); *f*: 9 (8–10); *h*: 33 (28–34). Distances between setae: *v*_1_–*v*_1_: 29 (26–30); *sc*_1_–*sc*_1_: 58 (49–59); *sc*_2_–*sc*_2_: 55 (50–60); *c*_2_–*c*_2_: 108 (95–109); *c*_1_–*c*_1_: 65 (56–68); *c*_1_–*c*_2_: 24 (20–23); *d*–*d*: 40 (34–40); *e*–*e*: 43 (41–44); *e*–*f*: 6 (6–7); *f*–*f*: 34 (33–34); *h*–*h*: 30 (27–30). Venter—lengths of setae: *1a*: 16 (15–20); *2a*: 18 (14–19); *3a*: 18 (16–21); *3b*: 20 (17–22); *3c*: 16 (13–19); *4b*: 20 (15–20); *ps*: 9 (7–9). Distances between setae: *1a*–*1a*: 8 (6–7); *2a*–*2a*: 29 (22–34); *3a*–*3a*: 16 (14–18); *3b*–*3b*: 38 (32–34); *3c*–*3c*: 40 (33–42); *4b*–*4b*: 21 (18–21); *ps*–*ps*: 17 (15–18). Length of PrP: 62 (55–64); width of PrP: 79 (85–104); ap. 1–1: 17 (14–17); ap. 2–2: 35 (34–40). Length of tegula: 23 (19–25); width of tegula: 30 (25–26). Leg segments and leg setae (lengths): Tbt I: 15 (15–16); *ω* I: 5 (4,5–5); *φ*_2_: 6 (5–7); *k*: 5 (6–7); *ω* II: 4 (4–5); *pl″* II: 7 (6–7); FeGe IV: 24 (21–25); Tbt IV: 8 (8–9); Fe *v′*: 13 (10–13); Ge *v′*: 26 (18–24); Tb *v′*: 48 (44–48); Ta *tc″*: 162 (140–174); Ti *v″*: 11 (9–11).

Male and larva: unknown.

Type material: holotype female and 11 paratype females found in galleries of *Scolytus* +/or *Chramesus* sp. (*Coleoptera*: *Curculionidae*) in *Lonchocarpus* sp. (Fabaceae), Chamela, State of Jalisco, Mexico, 12/X/1982, coll. Atkinson & Equihua (sample No 5-074). Holotype female and nine female paratypes deposited at CNC; one female paratype deposited at AMUNC.

Etymology: the species is named *jaliscoe* after the Mexican state of its origin.

Remarks: This mite was found inside the galleries of scolytid beetles. The collectors did not know if they were made by a *Scolytus* or *Chramesus* spp. The species of *Scolytus* reported on *Lonchocarpus* trees is *S. propinquus* Blandford. There are four different species of *Chramesus* associated with *Lonchocarpus* (S.L. Wood, personal communication, Brigham Young Univ., Provo). Two other phoretic females species of Heterostigmatina in the same sample were collected in association with these bark beetles; they represent an undetermined species of the pygmephorid genus *Elattoma* (fungivores) and the acarophenacid genus *Paracarophenax* (parasitoids) (E.E. Lindquist, personal communication, Agriculture Canada, Ottawa).

## 4. Discussion

**Figure 7:**
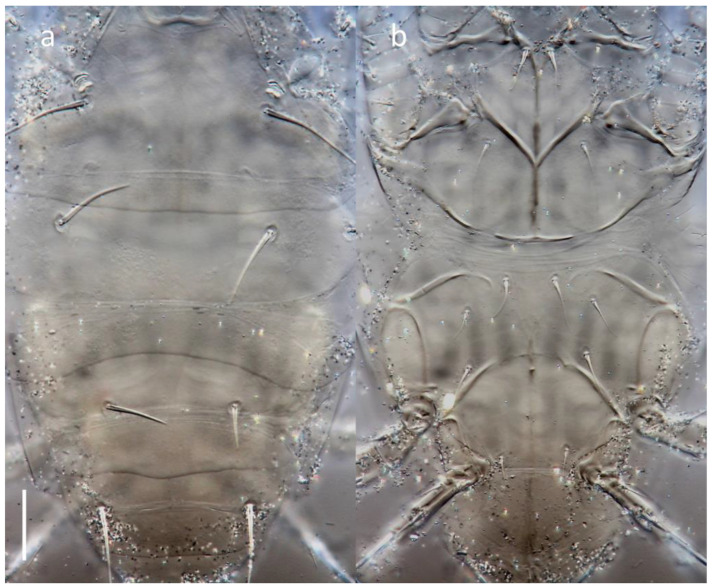
*Ochyronemus jaliscoe* sp. n. female idiosoma revealing plications of pleurae, DIC; (**a**) dorsal aspect, (**b**) ventral aspect. Scalebar is 20 µm.

Morphology: In general, the new genus shares many morphological similarities with its nearest kin genera, but in a somewhat mosaic manner. Some of these characters have either not been reported or are difficult to interpret. The idiosoma has distal transverse fine striations separating tergites C, D, EF, and H. Other such areas exist on the venter between the propodosomal and metapodosomal plates, including beneath the base of the tegula (Figure 7). There is no indication as to the function of these plications, but a reasonable hypothesis is that they serve to allow for the distension of the posterior idiosoma known as physogastry. Such a phenomenon is already known for another tarsonemid associated with bark beetles, i.e., *Iponemus* Beer & Nucifora [8]. However, it has not been demonstrated for the *Pseudotarsonemoidini* branch so far, and particularly *Tarsobisulcus,* which has similar plications (see Figure 6E in [19]), but distension has not been observed. On the other hand, a genus of the same subfamily (though of another tribe, *Tarsonemellini*), *Ficotarsonemus* Ho, has been reported with such development.

**Figure 8:**
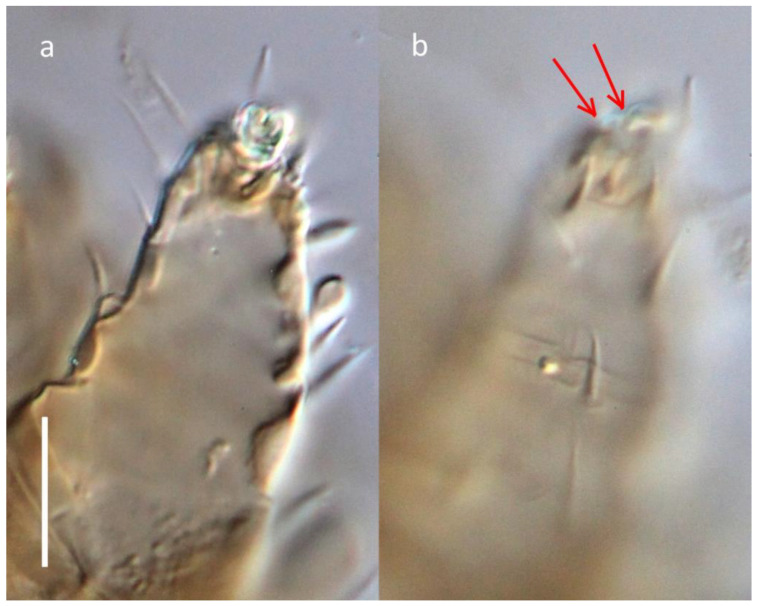
*Ochyronemus jaliscoe* sp. n. female leg I claw, DIC; (**a**) near-basal aspect showing split, (**b**) near-distal aspect with red arrows pointing at distal ends of claw split. Scalebar is 10 µm.

Another odd feature of this new taxon is the condition of the claw on tarsus I. Phylogenetically, *Ochyronemus* n. gen. is flanked by two genera, showing a spectrum of variability: most of *Pseudotarsonemoides* have their claw prominent, well-curved, and unitary (with one minor deviation in *P. longisetus* Khaustov, see [19] for comments), while the sole member of *Tarsobisulcus* has its claw split along its length distally. In *Ochyronemus,* however, this character is not uniform and needs further study. Several specimens display an incomplete division of the basal part of claw (Figure 8a), resulting in the asymmetric terminal bipartition of the claws’ blade (Figure 8b; red arrows). Notably, the claw of the holotype female is not partitioned. Two explanations can be proposed: the split is universal to all females but can be seen only when the claw is not depressed or bent downward. Alternately, this feature is variably manifested among individuals, i.e., there are females with their claw split (in varying measure) or whole (solid). Unfortunately, the lack of live specimens associated with their hosts did not allow for the use of Scanning Electron Microscopy to resolve these questions.

Systematic position and phylogeny: Following Lindquist [8], *Ochyronemus jaliscoe* gen. n. sp. n. is ultimately a member of the subfamily Pseudotarsonemoidinae by sharing the following attributes, as presented in the key to its parent group: “metapodosomal venter with 3 or usually 4 pairs of setae, including 1 pair between bases of legs IV in female” […] “leg I with membranous part of ambulacrum reduced or absent, and with large, nearly sessile claw” (quoted from [8]). The subfamily comprises two tribes, *Pseudotarsonemoidini* and *Tarsonemellini*, and the new genus is assignable to the *Pseudotarsonemoidini* due to its sharing of the following character states: cheliceral stylets not occupying the entire length of the gnathosomal capsule when retracted, the metapodosomal ventral plate always having four pairs of setae (*3a*, *3b*, *3c*, and *4b*), and the dorsum with a pair of capitate sensilli *sc*_1_. The genus *Ununguitarsonemus* Beer & Nucifora is considered immediately related to the *Pseudotarsonemoides*-*Tarsobisulcus*-*Ochyronemus* cluster due to it sharing the presence of an enlarged hooked claw (though rarely sessile) on tibiotarsus I in females and seta *pl″* on tarsus II of all instars. The new genus can be included in the one cluster with *Pseudotarsonemoides-Tarsobisulcus* branch due to the presence of a tonguelike, enlarged tegula, reduced posteromedial apodeme that is not bifurcate anteriorly; absent ventral setae *ag*; distinctly stout and robust tibiotarsus I. The *Pseudotarsonemoides* diagnosis [8] identifies some apomorphic character states as its key determinants, which are not observed in the new genus and *Tarsobisulcus*: the horseshoe shape of the pharynx, coupled lateral and medial emarginations of the dorsal plates D and EF, bilobate anterior margin of metapodosomal ventral plate (latter two unique against all other genera of Tarsonemidae), and a reduction in the number of setae on femora I and II. These morphological traits can be regarded as defining synapomorphies of the *Pseudotarsonemoides* genus. Two derived character states position the new genus closer to *Pseudotarsonemoides* than *Tarsobisulcus*, namely, the hoodlike prodorsum, extended over at least half the length of the gnathosoma, and very short setae *f* inserted closely posteromediad to the much longer *e*.

The new genus *Ochyronemus* presents some apomorphic character states unseen in the previously known species of this cluster; these are long and thin chelicerae occupying approximately half of the gnathosomal length, tibiotarsus I being relatively as long as it is wide at the base, and a tibial sensory group deprived of solenidion *ϕ*_2_. The latter character state is not unique since it has been reported in *Pseudotarsonemoides peruviensis* [18]. This condition, however, may be the homoplasy achieved independently.

The key to females of genus-level taxa of the tribe *Pseudotarsonemoidini* (couplets 1, 2, 3, 5, and 6 are derived from [8] with minor alterations):

1. Leg I with claw tightly hooked basally; tibiotarsus I with unguinal setae well developed in apposition to apex of claw. Spinelike seta Ta II *pl″* present near solenidion ω. …………………………………………………………………………………………………2

- Leg I with claw gently hooked basally, or claw absent; tibiotarsus I with unguinal setae reduced, not in apposition to claw. Spinelike seta Ta II *pl″* absent beside ω …………………………………………………………………………………………………5

2. Pharynx heavily sclerotized, distinctively horseshoe-shaped; anterior margin of metapodosomal venter doubly arcuate, deeply bilobate. Fe I with three setae, Fe II with two setae, and femoral part of Fege III with one seta …………… *Pseudotarsonemoides*

- Pharynx variously formed, but not horseshoe-shaped; anterior margin of metapodosomal venter not deeply bilobate. Fe I with four setae, Fe II with three setae, femoral part of Fege III with two setae ………………………………………………………3

3. Tegula enlarged, tonguelike, covering much of aggenital plate; setae *ag* absent; prodorsal shield well extended, hoodlike, over at least half of gnathosoma, also covering stigmata which open on ventral surface of shield…………………………………4

- Tegula usually not covering much of aggenital plate (if so, then elongate-triangular, not tonguelike in outline); setae *ag* present; prodorsal shield somewhat hoodlike over gnathosoma, but not covering stigmata which open dorsally on edge of shield ……………………………………….……………………………………*Ununguitarsonemus*

4. Anterior margin of metapodosomal ventral plate concave, entire; tibiotarsus I ca. as long as wide (terminal claw not included) without tibial solenid *φ*_2_ ……*Ochyronemus*

- Anterior margin of metapodosomal ventral plate protruding mid-anteriad in form of triangular projection; tibiotarsus I ca. 1.3x longer than wide with tibial solenid *φ*_2_ present ………………………………………………………………………………*Tarsobisulcus*

5. Legs II and III with symmetrically vestigial claws; tibial region of leg I with five setae in addition to sensory cluster, *l″* present; prodorsal shield not covering stigmata which open on dorsolateral edge of shield slightly anteriad of vertical setae; dorsal setae collectively slender, the scapular pair longer than any of the tergital pairs; leg IV with two setae on tibiotarsus……………………………………*Polyphagotarsonemus*

- Legs II and III with claws well developed or asymmetrically reduced, the posterior member usually vestigial or absent on leg III, and sometimes on leg II, of adult female; tibial region of leg I with four setae in addition to sensory cluster, *l″* lacking; prodorsal shield covering stigmata which open on ventral surface of shield at level posterolaterad of vertical setae; dorsal setae collectively stout, the scapular pair similar in length to the tergital pairs; leg IV with three setae on tibiotarsus ………………………………6

6. Genu I with three setae; gnathosomal capsule and palpi prolonged anteriorly, conspicuously beaklike; leg I with well-developed claw; dorsal shielding unornamented; gnathosoma deeply invaginated in podosoma, and supported ventrally by anteromedian projection of coxisternal plates I ………………………………*Nasutitarsonemus*

- Genu I with four setae; gnathosomal capsule not beaklike, with palpi short; leg I lacking a claw; dorsal shielding lineate or reticulate; gnathosoma not invaginated in podosoma, and lacking a ventral support formed by coxisternal plates I …*Tarsanonychus*

## Data Availability

The original contributions presented in this study are included in the article. Further inquiries can be directed to the corresponding author.
